# Chemical Characterization, Antioxidant, and Anticholinesterase Activities of *Libidibia ferrea* (Mart. Ex Tul.) L.P.Queiroz and In Silico Studies with the Acetylcholinesterase Enzyme

**DOI:** 10.1002/cbdv.202500550

**Published:** 2025-05-22

**Authors:** Lucas Soares Frota, Sara Ingrid Caetano Gomes Barbosa, Camila Costa Feitosa, Francisco Flávio da Silva Lopes, Daniel Pereira de Oliveira, Leonardo Soares Freitas, Adriano Lincoln Albuquerque Mattos, Wildson Max Barbosa da Silva, Hamilton Mitsugu Ishiki, Selene Maia de Morais

**Affiliations:** ^1^ Postgraduate Programme in Natural Sciences, Natural Products Chemistry Laboratory State University of Ceará Fortaleza Brazil; ^2^ Postgraduate Programme in Biotechnology, Natural Products Chemistry Laboratory State University of Ceará Fortaleza Brazil; ^3^ Veterinary medicine course, Natural Products Chemistry Laboratory State University of Ceará Fortaleza Brazil; ^4^ Embrapa Tropical Agroindustry Fortaleza Brazil; ^5^ State University Vale do Acaraú, Chemistry Course Sobral Brazil; ^6^ Center for Education, Science and Technology in the Inhamuns Region State University of Ceará Fortaleza Brazil; ^7^ Chemistry Course, Natural Products Chemistry Laboratory State University of Ceará Fortaleza Brazil

**Keywords:** acetylcholinesterase, antioxidant, essential oil, *Libidibia ferrea*, spectroscopy

## Abstract

*Libidibia ferrea* is used in many parts of Brazil for a variety of conditions, such as inflammation, respiratory disorders, muscle pain, and gastrointestinal problems. This study evaluated the chemistry and antioxidant and acetylcholinesterase (AChE) inhibitory activities of *L. ferrea* extracts. Hydroethanolic extracts of leaves (ELLF) and bark (EBLF) were obtained by maceration, while a methanolic fraction of seeds (MFLF) was prepared by Soxhlet extraction and essential oil of flowers (EOLF) extracted by hydrodistillation. Antioxidant activity was evaluated by scavenging 2,2‐diphenyl‐1‐picrylhydrazyl and 2,2'‐azino‐bis(3‐ethylbenzothiazoline‐6‐sulfonic acid) radicals, and AChE inhibition using the Ellman method. The ELLF, EBLF, and MFLF were characterized by high‐performance liquid chromatography showing the presence of gallic acid, chlorogenic acid, catechin, caffeic acid, rutin, and ellagic acid. EOLF and MFLF showed high tannin content and exhibited antioxidant and AChE inhibitory effects similar to standards. Molecular docking revealed that (Z)‐9‐tetradecenyl acetate, the major compound of EOLF identified by gas chromatography/mass spectrometry, interacts with AChE at the same site as galantamine. These findings suggest that *L. ferrea* has promising therapeutic potential for treating acetylcholine‐related diseases such as Alzheimer's.

## Graphical abstract

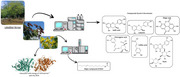

## 1 Introduction

*Libidibia ferrea* (Mart. Ex Tul.) L.P.Queiroz, formerly known as *Caesalpinia ferrea* is a species of tree native to Brazil, belonging to the Fabaceae family. In Brazil, this species is known by several common names, such as Jucá, Pau‐ferro, Pau‐ferro‐verdadeiro, Pau‐ferro‐do‐sertão, among others. This tree is widely distributed in tropical and subtropical regions of Brazil, being found mainly in the Caatinga and Cerrado biomes. *L. ferrea* is used in many parts of Brazil for a variety of conditions, such as inflammation, respiratory disorders, muscle pain, and gastrointestinal problems [1, 2]. The bark, roots, and leaves of the plant are often used in the form of teas, extracts, and other herbal products [3]. In the Amazon region of Brazil, the fruits of *L. ferrea* are widely used as an antimicrobial mouthwash in oral infections, through the preparation of an alcoholic tincture with dried fruit powder [4].

*L. ferrea* is not present on the list of Brazilian flora species threatened with extinction, according to Ordinance 443/2014, from the Ministry of the Environment, published in the Official Gazette of the Union on December 18, 2014 [5]. The species is also not included on the red list of those threatened with extinction, published by The IUCN Red List of Threatened Species 2019 [6]. Then *L. ferrea* is a useful and available medicinal plant for rational use to produce phytotherapics.

Previous phytochemical studies have reported the presence of bioactive secondary metabolites such as gallic acid, ellagic acid, catechin, epicatechin, quercetin, and Pauferrol A in different parts of *L. ferrea*, known for their potential to treat/inhibit Alzheimer's disease (AD) [3, 7].

AD is a neurodegenerative disease that affects thousands of people around the world. Acetylcholinesterase (AChE) is the crucial enzyme in the hydrolysis of one of the best‐known neurotransmitters, acetylcholine (ACh), which has been associated with the pathophysiology of AD. Therefore, AChE inhibition has been a widely used treatment strategy for AD. Several natural products have been investigated around the world with the aim of discovering new anticholinesterase agents that can be used as a therapeutic option in AD treatment [8, 9].

Free radicals are metabolic byproducts that are necessary for physiological function but can be toxic at high levels. These radicals increase gradually throughout the lifespan, impairing mitochondrial function and damaging all parts of the body, particularly the central nervous system. Several evidences suggest that oxidative stress may be one of the key mechanisms causing cognitive aging and neurodegenerative diseases such as AD. The most common means of reducing oxidative stress is antioxidant treatment [10].

Growing evidence has shown that inflammation could be a hallmark contributor to AD development and exacerbation. Pro‐inflammatory cytokines like tumor necrosis factor‐*α*, interleukin (IL)‐1β, and IL‐6 are upregulated in the brains of individuals with AD, which leads to an accumulation of Aβ plaque aggregates and tau hyperphosphorylation resulting in neuronal loss. Inflammation and AD are inevitably linked, with more research needed to solidify the mechanisms associated with them, including the intake of antioxidants for a healthier life [11].

The previous anti‐inflammatory and antioxidant activities of *L. ferrea* have driven the present study to evaluate several ethanol extracts and essential oils of the plant parts, their composition, and the activities of inhibition of AChE, for investigation of the neuropharmacological potential of *L. ferrea*, especially considering the growing interest in natural products with action on the cholinergic system, associated with the prevention and/or treatment of neurodegenerative diseases, such as Alzheimer's. Then, the aim of this study was to characterize chemical compounds in the *L. ferrea* extracts with antioxidant and anticholinesterase actions, which can be useful for AD treatment.

## 2 Results and Discussion

In the phytochemical prospecting test, all the extracts evaluated (ELLF, EBLF, and MFLF) presented a bluish color accompanied by the formation of a precipitate, indicating the presence of hydrolyzable tannins.

**FIGURE 1:**
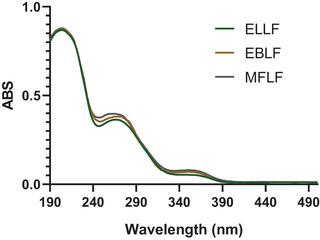
Ultraviolet (UV) spectrum for tannin‐rich extracts.

In the analysis of the ultraviolet (UV) spectrum of extracts (Figure 1), absorption peaks were identified at wavelengths between 260 and 280 nm, typical ranges associated with hydrolyzable tannins, evidencing that these compounds are predominantly present. Absorption in this range is attributed to electronic transitions π→π*, which occur in the aromatic rings characteristic of the structure of tannins [12].

**FIGURE 2:**
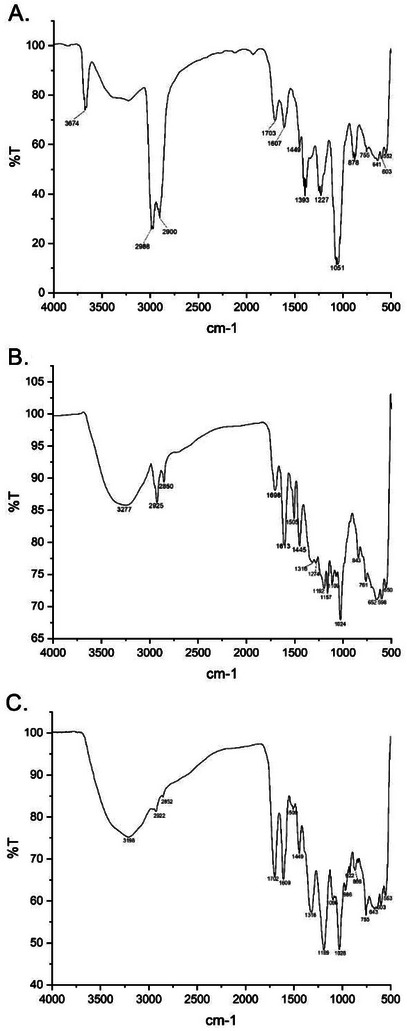
Fourier transform infrared spectroscopy (FTIR) for: (A) Hydroethanolic extract of the leaves of *L. ferrea* (ELLF); (B) Hydroethanolic extract of the bark of *L. ferrea* (EBLF); (C) Methanolic fraction of *L. ferrea* seeds (MFLF).

Hydrolyzable tannins are phenolic compounds widely found in plants and present characteristic bands in Fourier transform infrared spectroscopy (FTIR), due to the presence of specific functional groups (Figure 2). Among the main absorptions in the extracts, the broadest and most intense bands stand out in the region of 3156–3574 cm⁻¹, corresponding to the stretching of the O─H bonds, which are present in the hydroxyls of the phenolic rings and in the glucose structure. In addition, the region between 2800–3000 cm⁻¹ exhibits characteristic bands of C─H stretching, associated with both the sp^2^ bonds of the aromatic rings and the sp^3^ bonds of the glucose structure. The presence of ester groups and carboxylic acids can be confirmed by an intense absorption close to 1700 cm⁻¹, attributed to the C ═ O stretching, which represents the ester formation between phenolic acids (such as gallic acid and ellagic acid) and glucose. The aromatic rings of hydrolyzable tannins also generate significant bands between 1500 and 1600 cm⁻¹, related to the stretching of C ═ C bonds, evidencing the polyphenolic nature of these compounds. In addition, the ester groups and phenolic alcohols present bands in the range of 1200–1300 cm⁻¹, due to the C─O stretching, while the C─OH groups of aromatic hydroxyls absorb at 1000–1100 cm⁻¹. Thus, spectroscopic analysis allows a better characterization of hydrolyzable tannins in plant extracts, contributing to the understanding of their chemical and biological properties [13].

Phenolic compounds are well known for their potent antioxidant properties, which allow them to neutralize free radicals and protect cells from oxidative damage [14]. In this study, the total phenolic content was remarkably high in both ELLF and MFLF, with values ​​of 554.94 ± 1.24 and 506.82 ± 1.14 GAE/g, respectively. Among these, tannins were particularly abundant in MFLF, comprising almost 95% of the total phenolic compounds (Table 1). This significant presence point up the importance of tannins as major contributors to the observed antioxidant activity.

**TABLE 1:** Evaluation of total phenols, flavonoids, and tannins of *L. ferrea* extracts.

Sample	Total phenols (mg GAE/g)	Flavonoids (mg QE/g)	Tannins (mg TAE/g)
ELLF	554.94 ± 1.24^a^	32.86 ± 0.10^a^	472.29 ± 1.52^a^
EBLF	395.45 ± 1.03^c^	13.18 ± 0.14^c^	247.63 ± 0.19^b^
MFLF	506.82 ± 1.14^b^	18.22 ± 0.80^b^	476.63 ± 0.86^a^

ELLF—Hydroethanolic extract of the leaves of *L. ferrea*; EBLF—Hydroethanolic extract of the bark of *L. ferrea*; MFLF—Methanolic fraction of *L. ferrea* seeds; GAE—Phenol equivalent in milligrams of gallic acid per extract gram; QE—Flavonoid equivalent in milligrams of quercetin per extract gram; TAE—Tannic acid equivalent in milligrams extract gram; Different lower‐case letters indicate statistically significant differences (*p* < 0.05, ANOVA followed by Tukey's test).

^a,b,c^ Refers to statistical comparisons within the group.

Furthermore, tannins are widely documented in the literature not only for their antioxidant properties [15] but also for their neuroprotective effects [16]. These bioactive properties highlight their potential as therapeutic agents in the prevention and treatment of disorders related to oxidative stress, including neurodegenerative diseases such as Alzheimer's.

All samples showed strong antioxidant activity, with MFLF demonstrating impressive 2,2‐diphenyl‐1‐picrylhydrazyl (DPPH) radical inhibition (IC_50_ 2.39 ± 0.10 µg/mL), statistically comparable to the gallic acid standard and is even more potent than the commercial antioxidant Trolox (Table 2). This potent activity is likely attributed to the high phenolic content observed in Table 1. The IC_50_ values, all below 50 µg/mL, further confirm the strong antioxidant potential of these extracts [17]. Notably, tannins, which constitute almost 95% of the total phenolic content in MFLF, are recognized as major contributors to this activity. Their ability to donate electrons and stabilize free radicals shows up their role as key drivers of the observed antioxidant effects [18]. When compared to other plant extracts from the Fabaceae family, *Dalbergia nitidula* exhibits an IC₅₀ value of 9.31 ± 2.14 µg/mL for DPPH radical scavenging, indicating an antioxidant activity similar to that observed for the ELLF extract. In contrast, species such as *Virgilia divaricata*, *Erythrina caffra*, and *Baphia racemosa* show IC₅₀ values greater than 100 µg/mL [19] and are therefore classified as having low antioxidant activity [17]. These findings demonstrate that not all species within the Fabaceae family possess high antioxidant potential, highlighting the importance of conducting individual assessments within the group.

**TABLE 2:** Antioxidant and anti‐acetylcholinesterase activities of *L. ferrea* extracts.

Sample	IC_50_ DPPH^●^ (µg/mL)	IC_50_ ABTS^+●^ (µg/mL)	IC_50_ AChE (µg/mL)
ELLF	9.44 ± 0.84^c^	12.70 ± 0.14^d^	17.74 ± 0.25^c^
EBLF	12.61 ± 0.52^d^	13.20 ± 0.24^e^	16.58 ± 0.76^c^
MFLF	2.39 ± 0.10^a^	7.02 ± 0.09^a^	8.30 ± 1.18^b^
EOLF	6.48 ± 0.25^b^	8.37 ± 0.81^b^	6.36 ± 0.75^a^
Gallic acid (Standard)	2.14 ± 0.27^a^	13.01 ± 0.03^e^	—
Trolox (Standard)	12.35 ± 0.05^d^	12.13 ± 0.07^c^	—
Galantamine (Standard)	—	—	5.82 ± 0.02^a^
Physostigmine (Standard)	—	—	6.68 ± 0.08^a^

ELLF—Hydroethanolic extract of the leaves of *L. ferrea*; EBLF—Hydroethanolic extract of the bark of *L. ferrea*; MFLF—Methanolic fraction of *L. ferrea* seeds; EOLF—Essential oil from *L. ferrea* flowers; IC_50_ ‐ Inhibition concentration for 50%; Different lowercase letters indicate statistically significant differences (*p* < 0.05, ANOVA followed by Tukey's test).

^a,b,c,d,e^ Refers to statistical comparisons within the group.

Some antioxidant compounds have chemical structures capable of interacting with the catalytic or anionic site of the enzyme AChE, inhibiting its activity through hydrophobic interactions, hydrogen bonds, or Van der Waals forces. In addition, oxidative stress is associated with cholinergic dysfunction and increased AChE activity. Thus, compounds with antioxidant action can attenuate oxidative damage by neutralizing reactive oxygen species (ROS), positively modulating the cholinergic system and, consequently, reducing the overexpression or hyperactivity of this enzyme [20]. This relationship appears to be present in jucá extracts, which demonstrated high AChE inhibitory capacity, with IC₅₀ values ​​below 20 µg/mL [21]. Among the samples evaluated, EOLF and MFLF stood out with IC₅₀ values ​​of 6.36 ± 0.75 and 8.30 ± 1.18 µg/mL, respectively. Notably, the performance of EOLF is noteworthy for presenting an inhibitory potency statistically comparable to that of the classical reference inhibitors, galantamine (5.82 ± 0.02 µg/mL) and physostigmine (6.68 ± 0.08 µg/mL) (Table 2), both widely used as therapeutic models in the treatment of AD. These results suggest that the essential oil leaf extract (EOLF) represents a promising natural source of bioactive compounds with potential application in the development of AChE inhibitors, reinforcing the pharmacological relevance of the investigated species.

In a broader context, when assessing the AChE inhibitory activity of Fabaceae species previously reported to exhibit strong antioxidant potential (IC₅₀ < 50 µg/mL), *Cenostigma pyramidale* (IC₅₀ = 7.04 ± 0.04 µg/mL) and *Caesalpinia pulcherrima* (IC₅₀ = 13.14 ± 0.01 µg/mL) have shown particularly high enzyme inhibition. In contrast, *Bauhinia forficata* (IC₅₀ = 29.2 ± 1.87 µg/mL) and *Hymenaea stigonocarpa* (IC₅₀ = 23.98 ± 0.11 µg/mL) demonstrated only moderate inhibitory activity (20 < IC₅₀ < 50 µg/mL) [17, 22]. These findings emphasize the considerable variability in AChE inhibitory capacity among Fabaceae species, even among those with notable antioxidant properties, underscoring the importance of conducting complementary bioactivity evaluations for pharmacological screening.

### 2.1 Chromatographic Analyzes by High‐Performance Liquid Chromatography and Gas Chromatography/Mass Spectrometry

The characterization and quantification of compounds, performed by high‐performance liquid chromatography‐diode array detection (HPLC‐DAD), revealed the presence of gallic acid, chlorogenic acid, catechin, caffeic acid, rutin, and ellagic acid in almost all parts of the plant (Table 3). The EBLF was the only extract to show the presence of quercetin, with 7.26 ± 0.60 mg/g. The ELLF had the highest concentration of ellagic acid, with 26.28 ± 0.47 mg/g of extract. Hydrolyzable tannins are phenolic compounds synthesized by plants through the shikimate and phenylpropanoid pathways, with β‐glucogallin as a central precursor.

**TABLE 3:** High‐performance liquid chromatography (HPLC) quantification of phenolic compounds in *L. ferrea* leaves, bark, and seeds.

Constituents	R_t_ (min)	Concentration (mg/g of extract)		
		ELLF	EBLF	MFLF
**Phenolic acids**				
Gallic acid	3.56	2.44 ± 0.78	2.08 ± 0.58	22.38 ± 1.74
Chlorogenic acid	3.78	6.58 ± 0.44	2.99 ± 0.73	7.78 ± 0.73
Caffeic acid	5.52	5.35 ± 0.82	—	2.30 ± 0.49
Ellagic acid	7.28	26.28 ± 0.47	22.58 ± 0.01	8.04 ± 0.58
**Flavonoids**				
Catechin	4.63	14.63 ± 0.30	3.45 ± 0.12	34.97 ± 0.72
Rutin	6.70	11.08 ± 0.13	2.30 ± 0.81	5.59 ± 0.67
Quercetin	23.03	—	7.26 ± 0.60	—

R_t_—Retention time; ELLF—Hydroethanolic extract of the leaves of *L. ferrea*; EBLF—Hydroethanolic extract of bark of *L. ferrea*; MFLF—Methanolic fraction of *L. ferrea* seeds.

The presence of gallic and ellagic acids identified in the extracts confirms the presence of hydrolyzed tannins since these are synthesized from gallic acid, via the shikimate pathway [23, 24, 25]. These compounds are also known for their antiviral, antiparasitic, anti‐inflammatory, antidiarrheal, and antioxidant activities [26].

MFLF and EOLF were the more promisor extracts as antioxidant and anticholinesterase inhibitors, then hydrolyzable tannins from methanol fraction of seeds were the most promising extract for Alzheimer's treatment, although ELLF and EBLF should also be considered since they are more accessible for collection throughout the year.

The extraction of the essential oil from *L. ferrea* had a yield of 0.02%. The gas chromatography/mass spectrometry (GC/MS) analysis of this oil (Table 4) revealed that (Z)‐9‐tetradecenyl acetate was identified as the main compound (59.70%). This substance is known for its characteristic aroma, often described as a floral, sweet, and fruity odor. In some insects, for example, this substance can act as an aggregation pheromone, attracting individuals of the same species to gather in certain places [27]. Notably, the identification of constituent in *L. ferrea* essential oil is unprecedented and, to the best of our knowledge, has not been previously reported in the literature.

**TABLE 4:** Gas chromatography/mass spectrometry (GC/MS) analysis of the essential oil from *L. ferrea* flowers.

Constituents	R_t_ (min)	KI_lit_	Yield (%)
2‐Methylpentanal	3.13	745	8.04
(E)‐1‐Methoxy‐3‐hexene	3.55	825	8.25
(E)‐3‐Hexen‐1‐ol	4.07	851	16.10
(Z)‐9‐Tetradecenyl acetate	38.35	1779	59.70
Hexadecyl butyrate	38.48	1978	7.91

KI—Kovats index literature; R_t_—Retention time.

The second constituent is (E)‐3‐Hexen‐1‐ol (16.10%), an organic compound produced by the plant that repels insects. The presence of volatile compounds or essential oils in plants provides an important plant defense strategy, especially against herbivore insect pests. These volatile compounds also play a vital role in plant‐plant interactions and serve as attractants for some insect pollinators [28]. (E)‐3‐Hexen‐1‐ol, called the leaf alcohol, is mainly used in the perfume industry as a fragrance, in the food industry as fruit and vegetable flavor, as well as in aromatherapy, and in medicine. It is used in a wide range of products because of its characteristic intense fresh green grass odor. The (E)‐3‐Hexen‐1‐ol is also identified as a pheromone for some insects, and it has the characteristic of attracting different predatory insects [29]. The essential oil from *L. ferrea* although showing good action as an antioxidant and anticholinesterase inhibitor, is produced in very low percentages, nevertheless the compounds present similarity to galantamine and physostigmine in mode of action and interaction energies, representing templates for organic synthesis, aiming more specific applications for DA treatment.

The presence of only five compounds in the essential oil of *L. ferrea* flowers (Table 4), one of which accounts for approximately 59.70% of the total composition, suggests a chemical profile characterized by the dominance of a major constituent. This pattern is frequently observed in essential oils obtained from flowers, which tend to present specific volatile compounds with ecological functions, such as attracting pollinators. The predominance of a single metabolite may be associated with the existence of chemotypes, genetic variations within the species that prioritize the biosynthesis of certain compounds, in addition to being influenced by seasonal and environmental factors, such as light and temperature at the time of collection [30, 31, 32]. Additionally, the hydrodistillation extraction technique may favor the recovery of more volatile and thermostable compounds, resulting in a profile with low chemical diversity detectable by GC‐MS [33, 34]. It is important to highlight that the absence of a greater diversity of detected compounds does not invalidate the biological activity of the oil, since the majority of constituents may be mainly responsible for relevant pharmacological properties.

### 2.2 Molecular Docking of Essential Oil Constituents in AChE

To validate the molecular docking protocol, the crystallographic structure of galantamine bound to human AChE (PDB ID: 4EY6) was redocked, yielding a root mean square deviation (RMSD) of 0.632. This low RMSD value confirms the high accuracy and reliability of the docking procedure [35]. The recalculated binding free energy for galantamine was −10.4 kcal/mol, which serves as a robust reference point for evaluating the binding affinities of other ligands.

**FIGURE 3:**
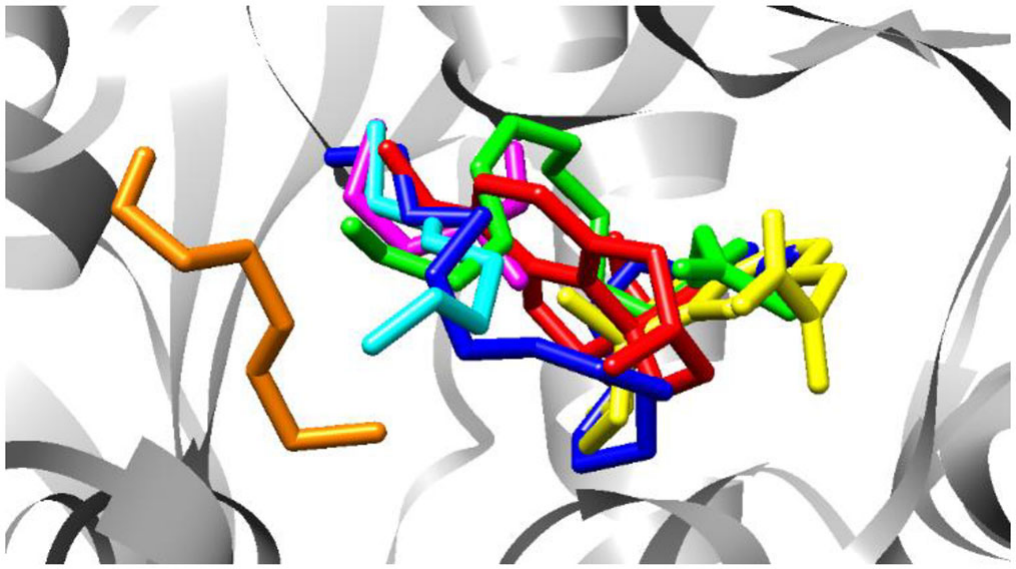
Molecular docking result indicating the position of galantamine in red, physostigmine in yellow, hexadecyl butyrate in blue, (Z)‐9‐tetradecenyl acetate in green, (E)‐1‐methoxy‐3‐hexene in cyan, 2‐methylpentanal in magenta, and (E)‐3‐hexen‐1‐ol in orange.

As presented in Table 5, the binding free energies of galantamine, physostigmine, hexadecyl butyrate, (Z)‐9‐tetradecenyl acetate, (E)‐1‐methoxy‐3‐hexene, 2‐methylpentanal, and (E)‐3‐hexen‐1‐ol were calculated using AutoDock Vina. Both galantamine and physostigmine exhibited strong binding affinities and engaged in interactions with key active site residues, particularly Gly120, Tyr124, Ser125, and Glu202. These residues appear to play a central role in ligand stabilization and, by extension, in AChE inhibition. The spatial distribution of these compounds within the AChE active site is illustrated in Figure 3, where their binding orientations and overlaps can be directly visualized.

**TABLE 5:** Binding free energy values calculated through molecular docking and the amino acid residues in the active site interacting with the ligands up to a distance of 4 Å.

Compound	AutoDock free binding energy	Interactions	
	(kcal/mol)	Hydrophobic	Hydrogen bonds
Galantamine (+)	−10.4	Trp86, Gly120, Gly121, Gly122, Tyr124, Ser125, Ser203, Phe295, Phe297, Tyr337, Phe338, His447	Glu202
Physostigmine (+)	−9.0	Tyr72, Asp74, Trp86, Asn87, Gly120, Gly121, Tyr124, Ser125, Gly126, Tyr133, Glu202, Tyr337, His447, Gly448	—
(Z)‐9‐tetradecenyl acetate	−6.8	Asp74, Trp86, Asn87, Gly120, Gly122, Phe297, Tyr337, Phe338, Tyr341, His447	Tyr124, Ser125
Hexadecyl butyrate	−6.5	Trp86, Gly120, Gly121, Gly122, Tyr124, Glu202, Ser203, Trp236, Phe295, Phe297, Tyr337, Phe338, Ile415, Hys447, Gly448	Tyr133
(E)‐1‐methoxy‐3‐hexene	−4.7	Gly122, Ser203, Trp236, Phe295, Phe297, Phe338	Tyr124
2‐methylpentanal	−4.6	Gly120, Ser203, Trp236, Phe338, Phe295, Phe297, His447	Gly121, Gly122, Ala204
(E)‐3‐hexen‐1‐ol	−4.6	Trp286, Val294, Phe295, Phe297, Tyr337, Phe338, Tyr341	Ser293, Arg296

Among the natural compounds evaluated, hexadecyl butyrate and (Z)‐9‐tetradecenyl acetate stood out, with binding free energies of −6.8 and −6.5 kcal/mol, respectively. Despite their lower affinities compared to the reference inhibitors, both compounds interact with critical active site residues. Specifically, hexadecyl butyrate forms hydrophobic interactions with Trp86, Gly120, Tyr124, and a hydrogen bond with Tyr133, while (Z)‐9‐tetradecenyl acetate establishes hydrophobic interactions with Asp74, Trp86, Phe297, and hydrogen bonds with Tyr124 and Ser125.

The biological relevance of these interactions is particularly notable for (Z)‐9‐tetradecenyl acetate, the major constituent (59.70%) of the essential oil from *L. ferrea* flowers. Although its docking score is modest when compared to galantamine, its in vitro IC₅₀ was statistically equivalent to that of the commercial drug (Table 2). This supports the hypothesis that the binding mode of (Z)‐9‐tetradecenyl acetate, which closely ressembles that of galantamine in terms of key residue engagement, may underpin its effective inhibition of AChE [36].

In contrast, other natural compounds such as (E)‐1‐methoxy‐3‐hexene, 2‐methylpentanal, and (E)‐3‐hexen‐1‐ol demonstrated weaker binding energies and limited interactions with the AChE active site. These ligands formed fewer hydrophobic and hydrogen bonds and lacked engagement with the critical residues identified above, suggesting a much lower inhibitory potential.

These findings underline the pharmacological potential of (Z)‐9‐tetradecenyl acetate and hexadecyl butyrate as promising natural AChE inhibitors. Their interactions with key catalytic residues and favorable docking profiles justify further investigation, especially considering the biological relevance demonstrated in in vitro assays for (Z)‐9‐tetradecenyl acetate.

## 3 Conclusions

The present study characterizes the phenolic compounds in the extracts through HPLC analysis from various parts of *L. ferrea*, mainly composed of hydrolyzable tannins. Both extracts have a high inhibition activity of the DPPH radical (IC_50_< 50 µg/mL) and of the enzyme AChE (IC_50_ < 20 µg/mL). In silico analysis revealed that the main compound of the essential oil identified in the GC, (Z)‐9‐tetradecenyl acetate, binds to the same active site as galantamine, further supporting its neuroprotective potential, especially in the context of neurodegenerative diseases such as AD.

Overall, the findings suggest potential therapeutic benefits from various parts of *L. ferrea* in treating AD through antioxidant and cognitive‐enhancing mechanisms. However, further studies are needed to validate these effects and assess their clinical applicability.

## 4 Experimental

### 4.1 Obtaining the Material

The leaves of the *L. ferrea* plant were collected in March 2023, at the State University of Ceará. A copy of this specimen is in the Prisco Bezerra Herbarium (EAC) of the Federal University of Ceará (UFC), with the botanical identification of *L. ferrea* (Mart. Ex Tul.) L.P. Queiroz was deposited under the number 66363 and identified by the botanist Hugo Pereira of Birth in March 2023. SisGen n° AFB0E62.

### 4.2 Extract of Leaves, Bark, and Seeds of *L. ferrea*

The leaves and bark of *L. ferrea* were collected (200 g), dried in an oven at 80°C, ground, and subjected to the maceration method with 70% ethanol in an ethanol‐material ratio of 1:1 at room temperature (25°C), for a period of 7 consecutive days, without renewal of the extracting liquid. The extracting liquid was then filtered and concentrated in a rotary evaporator at a temperature of 50°C. The solution was subjected to lyophilization, producing the ELLF with a yield of 10.48% and the EBLF with a yield of 12.31%.

The seeds of *L. ferrea* were collected (200 g), dried, ground, and submitted to the Soxhlet extraction method for 8 h with P.A. hexane and then for 8 h with P.A. methanol. The extracting liquid was then filtered and concentrated in a rotary evaporator at a temperature of 50°C. The polar solution of interest was subjected to lyophilization, producing the MFLF with a yield of 9.5%.

### 4.3 Obtaining Essential Oil From the Flowers of *L. ferrea*

The flowers of *L. ferrea* were collected (500 g) and extracted by hydrodistillation in a Clevenger‐type apparatus. For this purpose, the fresh flowers were added to a 1 L round bottom flask, together with distilled water until the flowers were completely covered inside the flask. The procedure lasted approximately 4 h after the start of steam condensation. The procedure lasted approximately 4 h after the start of vapor condensation. The oil was removed from the Clevenger apparatus and filtered with anhydrous sodium sulfate (to remove water).

### 4.4 Analysis of Tannins in Plant Extracts

To identify the types of tannins present in the plant extracts, qualitative phytochemical prospecting was initially performed using the ferric chloride reagent (FeCl₃) [37]. The extracts were then subjected to UV‐visible spectrophotometric analysis in scanning mode (200–800 nm), allowing the characteristic spectrum of the compounds present to be obtained [38].

### 4.5 Quantification of Total Phenols, Flavonoids and Tannins

The determination of total phenol content was performed using visible region spectroscopy with the Folin‐Ciocalteu reagent, following the methodology of Sousa et al. [39] For the quantification of flavonoids, a 2.5% aluminum chloride (AlCl_3_) reagent was used, following the methodology of Funari and Ferro [40]. The determination of total tannin content was carried out using visible region spectroscopy with the Folin‐Denis reagent [41].

### 4.6 Evaluation of Antioxidant Activities by DPPH and ABTS Free Radical Inhibition Methods

The antioxidant activity was evaluated using the DPPH method following the methodology described by Becker et al. [42] with modifications, and the 2,2'‐azino‐bis(3‐ethylbenzothiazoline‐6‐sulfonic acid) method as described by Rhee et al. [43] Both tests were conducted in a 96‐well flat‐bottom microplate using a BioTek Elisa reader, model ELX 800.

### 4.7 In Vitro Evaluation of AChE Inhibition

The inhibitory activity of the enzyme recombinant AChE was measured in 96‐well flat‐bottom plates using a BioTek ELISA reader, model ELX 800, with the “Gen5 V2.04.11” software, based on the methodology described by Re et al. [44] and Trevisan et al. [45] All solutions were used as negative standards, except the sample. All samples were analyzed in triplicate.

### 4.8 Fourier‐transform IR Spectroscopy

The determination of functional groups was performed by infrared spectroscopy, using an FTIR spectrophotometer FTIR Agilent FTIR (Cary 660 model) from 4000 to 400 cm^−1^ with a resolution of 4.0 cm^−1^ and 25 scans.

### 4.9 HPLC with DAD

To identify phenolic compounds, a methanolic solution of each sample (20 µL/mL) was injected into the HPLC system. Standards were obtained from Sigma, and analytical‐grade solvents were used for extraction, while HPLC‐grade solvents were used in the analyses. Samples and solutions were filtered through nylon membranes (0.45 and 0.22 µm) before analysis, which was performed in triplicate. HPLC‐DAD analysis was conducted using a Shimadzu system with a Shim‐pack ODS GOLD reversed‐phase column. The mobile phases were acetonitrile and Milli‐Q water acidified to pH 2.8, using a gradient: 0–15 min (20:80 v/v), 17–25 min (40:60 v/v), and 25–40 min (20:80 v/v). The flow rate was 1.0 mL/min, with a 20 µL injection volume and detection at 350 nm. Peaks were confirmed by retention time and DAD spectra (200–400 nm).

### 4.10 CG/MS Analysis of the *L. ferrea* Essential Oil

The essential oil of *L. ferrea* flowers was analyzed using a Shimadzu QP‐2010 equipment with an Rxt‐5MS capillary column (30 m × 0.25 mm × 0.25 µm), using helium as the carrier gas at a flow rate of 24.2 mL/min. The injector and detector temperatures were set to 250°C and the temperature program started at 35°C, increased at 4°C/min to 180°C, then at 17°C/min to 280°C, holding for the last 10 min. Mass spectra were obtained by electron impact (70 eV) and compared to the NIST library for compound suggestions. Identification was confirmed by comparing mass spectra and Kovats index with the NIST and Adams [46] databases. Experimental Kovat Index values were calculated through linear regression of retention times.

### 4.11 In Silico Study

The recombinant human AChE receptor (PDB: 4EY6) complexed with galantamine was obtained from the RSCB Protein Data Bank, with a resolution of 2.40 Å [47]. Water molecules and other compounds were removed, and polar hydrogen atoms and Kollman atomic charges were added. The chemical structure of galantamine was extracted from the corresponding PDB data, while the 2D structures of other ligands were drawn using ChemSketch, and their 3D structures were prepared with 3D Viewer. To confirm docking accuracy, the native ligand (galantamine) was removed, and re‐docking was performed with an RMSD < 2 Å considered successful. The RMSD between crystallographic and docked structures was calculated using the DockRMSD tool [48]. The calculations consider the formation of hydrogen bonds, attractive van der Waals interactions, electrostatic interactions, and attractive hydrophobic interactions [49]. Molecular docking was then performed using AutoDock 4.2 software to predict ligand orientations and calculate binding free energy [50]. The receptor was kept rigid, while the ligand was flexible, with the Lamarckian genetic algorithm as the search parameter. Each ligand was docked in 50 conformations within a 60 × 60 × 60 Å grid box. The results were analyzed with AutoDock Tools and visualized with Chimera [51]. The most energetically favorable docking poses were selected, and interactions were validated using Ligplot+ to generate 2D diagrams of hydrogen bonding and hydrophobic interactions [52].

## Author Contributions

The authors declare individual contributions to the article as follows: data collection, analysis, and the preparation of the manuscript. **Lucas Soares Frota**: conceptualization. writing – original draft writing – review and editing. **Sara Ingrid Caetano Gomes Barbosa**: methodology. **Camila Costa Feitosa**: methodology. **Francisco Flávio da Silva Lopes**: methodology. **Daniel Pereira de Oliveira**: methodology. **Leonardo Soares Freitas**: methodology. **Adriano Lincoln Albuquerque Mattos**: methodology. **Wildson Max Barbosa da Silva**: methodology. **Hamilton Mitsugu Ishiki**: methodology. **Selene Maia de Morais**: conceptualization. funding acquisition writing – review and editing. The completed manuscript underwent comprehensive review by all authors, resulting in approval for publication. All authors have read and agreed to the published version of the manuscript.

## Conflicts of Interest

The authors declare no conflicts of interest.

## Data Availability

The data that support the findings of this study are available from the corresponding author upon reasonable request.
